# Potential Properties of Plant Sprout Extracts on Amyloid *β*

**DOI:** 10.1155/2016/9347468

**Published:** 2016-06-27

**Authors:** Mizue Okada, Yoshinori Okada

**Affiliations:** ^1^Nutrition Section, Ageing and Nutrition Research, Yms Laboratory, Gifu 503-1306, Japan; ^2^Laboratory on Ageing & Health Management, Graduate School of Nursing & Health, Aichi Prefectural University, Tohgoku, Kamishidami, Moriyama, Nagoya 463-8502, Japan

## Abstract

The aim of this study is to examine the amyloid *β* (A*β*) inhibition mechanism of plant sprouts' aqueous extracts (PSAE). In this study, we screened the effects of five plant sprouts' extracts on A*β* (1–42) structure modification using gel electrophoresis. In PSAE, no band of A*β* monomer was recognized in Japanese butterbur. Similarly, the A*β* monomer band became light in buckwheat, red cabbage, broccoli, and brussels. The neuroprotective effects of PSAE were evaluated by measuring levels of A*β* in mixtures (A*β*  and PSAE) with A*β* ELISA assay. The treatment with PSAE decreased A*β* levels. The results indicated that the levels of red cabbage, Japanese butterbur, and broccoli were 9.6, 28.0, and 44.0%, respectively. The lowest value was observed with buckwheat. Furthermore, we carried out a Congo Red (CR) and A*β* binding experiment of PSAE to confirm the modification mechanism of PSAE. The correlation coefficient for the absorption spectrum peak of CR was found to be bigger than 0.8 (*r* = 0.882) which proved that the A*β* levels could be attributed to the peak of CR. In conclusion, we demonstrated that treatment with PSAE effectively decreases A*β* concentration. Thus, the mechanism that decreased the A*β* levels may be modification by PSAE.

## 1. Introduction

Alzheimer's disease (AD) [1] is currently the most lethal neurodegenerative disorder known. It is characterized by progressive neuronal loss and neuroinflammation in the brain. Neuropathology detects neuronal loss in association with the deposition of amyloid plaques. The aggregation of amyloid *β* (A*β*) peptides starts with changes in their secondary structure leading to *β*-sheet formation, progresses with aggregation of the misfolded peptides into oligomers, and culminates in the production of amyloid fibres that precipitate into the brain forming amyloid plaques. A*β* can be neurotoxic by a mechanism linked to peptide fibril formation. Therefore, A*β* peptides are very important in the research of AD. However, the mechanism by which A*β* produces brain dysfunction in patients with AD is largely unknown.

Sprouts are a source of various biologically active phytomolecules, including phenolic compounds, flavonoids, and vitamins [2, 3]. The health properties of sprouts depend on their phenolic compounds [4], which have interesting pharmacological properties. Much research has assessed the dietary role of polyphenolic substances and their characteristics, metabolic pathways, and biological effects [5]. Sprouts have been widely used to scavenge reactive oxygen species (ROS) and treat a variety of diseases [6]. We propose that extracts of sprouts have significant antioxidant activity and suspect the extract might be useful in preventing AD.

Złotek et al. proposed that the glycation process might contribute to both extensive protein cross-linking and oxidative stress in AD [7]. Nonenzymatic protein glycation is an endogenous process in which reducing the sugars that react with amino groups in proteins through a series of Maillard reactions forming reversible Schiff-base and Amadori compounds produces a heterogeneous class of molecules, collectively termed advanced glycation end products (AGEs) [8, 9]. A previous study discussed the globular amyloid-like deposits of D-ribose glycation of bovine serum albumin (BSA) aggregates. The amyloid-like aggregation of glycated BSA induces apoptosis in the neuronal cell. D-ribose saccharifies BSA which then misfolds rapidly and forms globular amyloid-like aggregations, which play an important role in cytotoxicity of neuronal cells [10]. In addition, glycation of A*β* markedly enhances its aggregation* in vitro*. Therefore, in the present study, we have investigated the effect of glycation on the aggregation pathways of BSA and lactalbumin (LAB). Although this reaction may not be directly related to AD, it is thought to be a good representative model of proteins that intrinsically evolve toward the formation of amyloid aggregates.

Although there are many reports on beneficial effects of plant food extracts to A*β*-induced neuronal cell death, their inhibition mechanisms are yet to be well understood. Therefore, study on the mechanisms is very important in the research of AD.

As a result of having examined modification to A*β* (1–42) by SDS-polyacrylamide gel electrophoresis of about 15 samples (5 sprouts × 3 extract methods) of the plant sprouts, we decided to focus on plant sprouts' aqueous extracts (PSAE) which showed the remarkable result. In addition, we experimented with A*β* alone and with a mixed sample (A*β*+PSAE). Using an amyloid *β* ELISA kit, we found that PSAE showed the inhibition effect on A*β*. Considering these findings, we examined the A*β*-inhibition mechanism of PSAE.

## 2. Materials and Methods

### 2.1. Preparation of Extracts from Plant Sprouts (PSE)

We selected five plant sprouts (buckwheat (*Fagopyrum esculentum*) sprout (BWS), red cabbage (*Brassica oleracea *var.* capitata*) sprout (RCS), broccoli (*Brassica oleracea* var.* italica*) sprout (BS), brussels (*Brassica oleracea* var.* gemmifera*) sprout (BRS), and Japanese butterbur sprout flower buds (*Petasites japonicus*) (JBB)). Plant sprouts were purchased from the market in Japan. Plant sprouts (100 g) were minced into 5 mm fragments and extracts were obtained with cold water (CW, 20°C), hot water (HW, 100°C, 10 min), and methanol (MH) overnight at room temperature (Table 1). The extracts were filtered. The MH filtrates were evaporated in a vacuum slightly below 40°C in a rotary evaporator. The MH extracts were prepared as a solution in dimethyl sulfoxide (DMSO). The collected filtrate was then stored at under −20°C until use. When used in assays, each sample was returned to ambient temperature, followed by filtration through a membrane filter (pore size 0.2 *μ*m).

### 2.2. Measurement of SDS-Polyacrylamide Gel Electrophoresis

As described previously [20], the A*β* modification by PSE was used with measurements of SDS-polyacrylamide gel electrophoresis (PAGE). Briefly, 75 *μ*L of PSE or H_2_O was combined with 75 *μ*L of A*β* (1–42) (10 *μ*M, Wako Pure Chemical Industries, Ltd.) solution. We allowed the mixture to sit for 20 h at 37°C before measuring SDS-PAGE. Ten *μ*L of incubated samples was mixed with 10 *μ*L of SDS-PAGE sample buffer and loaded on 15% SDS-polyacrylamide gel. The samples were electrophoresed at 40 A for 1 h. The gels were stained for protein with Quick CBB PLUS (Wako Co. Ltd). Molecular masses of the bands obtained were calculated with the help of the standard molecular weight markers (Precision Plus Protein*™* Prestained Standards, Bio-Rad Laboratories).

### 2.3. Determination of Total Phenolic Content (TPC)

We measured total phenolic content (TPC) with a modified version of the Folin-Ciocalteu method [21] using 0–0.1 mg/mL catechin as a standard. Briefly, 100 *μ*L of sample or standard was combined with 100 *μ*L of Folin-Ciocalteu reagent and 100 *μ*L of 2% Na_2_CO_3_ solution. We allowed the mixture to sit for 60 min before reading absorbance at 750 nm using an Ultrospec Visible Plate Reader II 96 (GE Healthcare Ltd., England) and calculating the concentration of plant sprouts' aqueous extracts (PSAE) as catechin equivalents per gram of plant sprouts.

### 2.4. 2,2-Diphenyl-1-picrylhydrazyl (DPPH) Radical Scavenging Activity

Scavenging of DPPH free radical is the basis of a common antioxidant assay. Therefore, we used the DPPH method. Employing the procedure described by Negro et al. [22], we measured the free radical scavenging activity of PSAE. After mixing a sample solution (100 *μ*L) with 100 *μ*L of 800 *μ*M DPPH-methanol solution and waiting for 30 min, we measured the absorbance of the sample solution at 520 nm against a blank. DPPH radical scavenging activity was exhibited as follows. The control ratio (%) was expressed as a percentage of the untreated control as follows: % control ratio = (A520 nm of PSAE treated DPPH-methanol solution/A520 nm of untreated control) × 100. All tests and analyses were run in triplicate and averaged.

### 2.5. Assessment of A*β* (1–42) Concentration

Levels of A*β* (1–42) in mixtures (10 *μ*M A*β* (1–42) 55 *μ*L and PSAE 55 *μ*L) were determined with human specific enzyme-linked immunosorbent assay (ELISA) (Wako, Osaka), according to the manufacturer's instructions. The mixtures (110 *μ*L) were added to microplate wells. The mixtures were then incubated at room temperature for 24 h. After 24 h incubation, we obtained 100 *μ*L of sample and distributed some of the sample into each well coated with a monoclonal antibody specific to the NH2-terminus region of human A*β*. Detected A*β* (1–42) was analyzed with a commercial kit according to instructions provided by the manufacturer (Wako Pure Chemical Industries). The detection limit of the assay was 0.1 pmol/L for A*β* (1–42). Epigallocatechin gallate (EGCG) was used as the positive control. The A*β* (1–42) level was measured as A450 nm. The control ratio (%) was expressed as a percentage of the untreated control as follows: % control ratio = (A450 nm of treated cells/A450 nm of untreated cells) × 100.

### 2.6. *In Vitro* Glycation of Bovine Serum Albumin (BSA) and Lactalbumin (LAB) Induced by D-Ribose

Inhibition of glycation was measured with a modified version of the Wei method [10]. After sterilization, using a Millex GV filter (Millipore, Cork, Ireland) to prevent bacterial growth, BSA and LAB were dissolved in 20 mM Tris-HCl (pH 7.4) to yield a stock solution of 20 mg/mL. D-ribose (1 M, a final concentration) was then prepared in Tris-HCl to final concentrations of 10 mg/mL BSA or LAB. PSAE were added to 20 mM Tris-HCl containing 1 M D-ribose and either BSA or LAB to acquire final concentrations of 10 mg/mL. EGCG was used as the positive control. Then, the solutions were incubated at 37°C for up to 24 h. After incubation, the fluorescent reaction products were assayed on a fluorophotometer (*λ*_ex_ 360 nm/*λ*_em_ 465 nm, multimode microplate reader Infinite F200, Tecan Trading AG, Switzerland). BSA or LAB, in the presence of D-ribose, was used as a control. The control ratio (%) was expressed as a percentage of the untreated control as follows: % control ratio = (fluorescence intensity of treated cells/fluorescence intensity of untreated cells) × 100. Each experimental condition was performed in triplicate.

### 2.7. *In Vitro* Aggregation of BSA and LAB Induced by D-Ribose

*In vitro* aggregation of BSA and LAB was measured by methods previously described [20]. Briefly, PSAE was added to 20 mM Tris-HCl containing 1 M (a final concentration) D-ribose and either BSA or LAB to acquire final concentrations of 10 mg/mL. The solutions were then incubated at 37°C for up to 24 h. After incubation, Thioflavin T (ThT, 30 *μ*M), commonly used to detect protein aggregations, was added to the solution to investigate whether any amyloid-like deposits formed at 37°C. After incubation for 10 min, the fluorescent reaction products were assayed on a fluorophotometer (*λ*_ex_ 430 nm/*λ*_em_ 465 nm). BSA or LAB, in the presence of D-ribose, was used as a control. In addition, EGCG was used as the positive control. The control ratio (%) was expressed as a percentage of the untreated control as follows: % control ratio = (fluorescence intensity of treated cells/fluorescence intensity of untreated cells) × 100.

### 2.8. Congo Red (CR) Binding Assay

The binding of CR was monitored using absorption spectroscopy. A 5.0 mM stock solution of CR was prepared in PBS. PSAE was added to PBS containing 10 mM (a final concentration) A*β* (25–35) and Congo Red (CR) solution to acquire final concentrations of 5.0 × 10^−6^ M. The solutions were then incubated at 25°C for up to 168 h. And then, the mixture was assayed on a UV-VIS spectrophotometer (Multiskan*™* GO Microplate Spectrophotometer, Thermo Fisher Scientific, MA, USA).

### 2.9. Statistical Analysis

We present all data as mean ± standard deviation of the three measurements. A statistical comparison between the groups was carried out using either ANOVA or Student's *t*-test. *P* < 0.05 were considered as statistically significant.

## 3. Results

### 3.1. Amyloid *β* Electrophoretic Analysis

**Figure 1:**
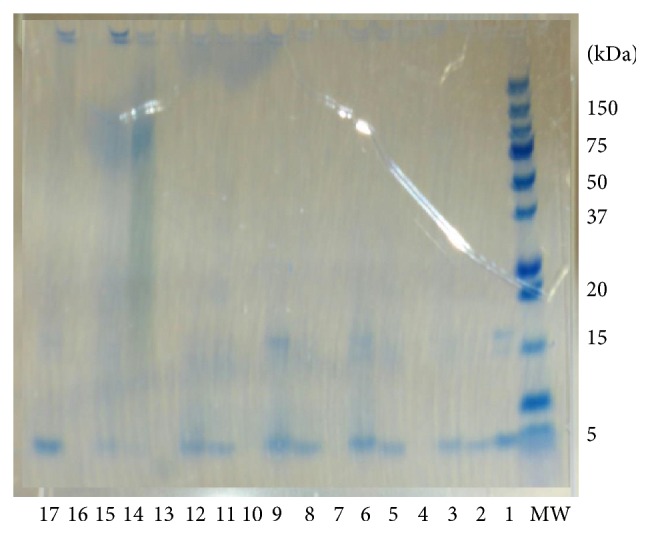
15% SDS-PAGE of the products of amyloid *β* (A*β*) (1–42) incubation with extracts from plant sprouts (PSE). SDS-PAGE illustrating the inhibition effect of PSE on A*β* (1–42). Lanes (from right to left) indicating the electrophoretic migration of A*β* (1–42) in the absence of PSE and in the presence of PSE. 1: A*β* (1–42); 2: cold water- (CW-) buckwheat sprout (BWS); 3: hot water- (HW-) buckwheat sprout (BWS); 4: methanol- (MH-) buckwheat sprout (BWS); 5: CW-red cabbage sprout (RCS); 6: HW-red cabbage sprout (RCS); 7: MH-red cabbage sprout (RCS); 8: CW-broccoli sprout (BS); 9: HW-broccoli sprout (BS); 10: MH-broccoli sprout (BS); 11: CW-brussels sprout (BRS); 12: HW-brussels sprout (BRS); 13: MH-brussels sprout (BRS); 14: CW-Japanese butterbur (JBB); 15: HW-Japanese butterbur (JBB); 16: MH-Japanese butterbur (JBB); 17: epigallocatechin gallate (EGCG). MW shows a molecular weight marker. All tested samples were analyzed by 15% Tris-HCl-SDS-PAGE, followed by Quick-CBB PLUS staining, as described in the experimental part.

In the present study, we investigated the effects of PSE on A*β* (1–42) structure modification. The mixture samples were electrophoresed at 40 A for 1 h (Figure 1). No band of A*β* monomer (4.5 kDa) was recognized in CW-JBB. Similarly, the A*β* monomer (4.5 kDa) band became light in CW-RCS, CW-BS, CW-BRS, and CW-BWS. These suggest that some special conformation, presumably in protein, was present in a major constituent of amyloid. Therefore, we carried out the following experiments involving PSAE that showed that its effect was remarkable in this electrophoretic experiment.

### 3.2. PSAE Reduces A*β* (1–42) Concentration

**Figure 2:**
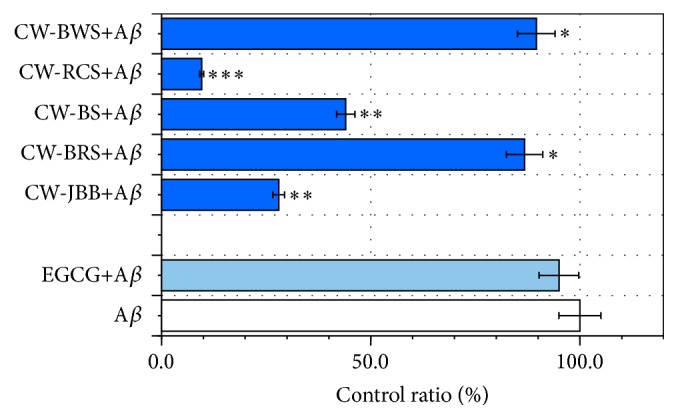
Inhibitory effects of plant sprouts' aqueous extracts (PSAE) on A*β* (1–42) concentration. Levels of A*β* (1–42) in mixtures (10 *μ*M A*β* (1–42) 55 *μ*L and PSAE 55 *μ*L) were determined with human specific enzyme-linked immunosorbent assay. The mixtures (110 *μ*L) were added to the microplate wells and then incubated at room temperature for 20 h. After 20 h incubation, A*β* (1–42) was analyzed with a commercial kit. Values are mean ± SD of the three measurements. ^*∗∗∗*^ *P* < 0.001, ^*∗∗*^ *P* < 0.01, and ^*∗*^ *P* < 0.05 compared with the controls.

To examine the effects of PSAE on the A*β* (1–42) concentration, PSAE mixture samples were analyzed by A*β* (1–42) ELISA.  Figure 2 illustrates that PSAE were associated with differential reduction in the levels of A*β* (1–42) (CW-RCS, 9.6%; CW-JBB, 28.0%; CW-BS, 44.0%; CW-BRS, 86.7%; CW-BWS, 89.6%), demonstrating that treatment with PSAE effectively decreases A*β* (1–42) concentration. CW-RCS treatment demonstrated the strongest A*β*-inhibition potential (Figure 2). These showed low values from 10 *μ*M EGCG (95.0%).

### 3.3. Relationships between Total Phenolic Content (TPC), DPPH Radical Scavenging Activity, and A*β* (1–42) Levels of PSAE

TPC was expressed as mg of catechin equiv/g of matter. Significant differences were observed for TPC among the 5 plant sprout varieties. The total phenolic content varied among species from 6.5 to 433.1 mg (Table 1). The aqueous extracts from broccoli sprouts (CW-BS) contained the lowest amounts. The aqueous extracts from buckwheat, red cabbage, and brussels sprouts contained roughly 1.5–2.5 times the total phenolic content of broccoli sprouts. The aqueous extracts from Japanese butterbur sprouts contained the highest amount of total phenolics (223.3 mg (+)-catechin equivalents/g). Broccoli sprouts contained 1/34 of the phenolics observed in Japanese butterbur sprouts.

The radical scavenging effect is proportional to the disappearance of DPPH in test samples. Based on this principle, the radical scavenging effect of each plant sprouts extract was measured. The extracts of 5 plant sprouts were compared for their radical scavenging activities against DPPH radical. With regard to the value, the highest radical scavenging activity was found in the extract of CW-BWS (25.0%) and the lowest activity was found in CW-RCS (100.6%). The order of the scavenging activity was 25.0, 55.6, 90.8, 91.0, and 100.6% for CW-BWS, CW-JBB, CW-BS, CW-BRS, and CW-RCS, respectively. However, the scavenging activity of all extracts except for CW-BWS was less than that of ±catechin (50.7%). The activity of CW-BWS was approximately 4 times higher than that of CW-RCS.

**Figure 3:**
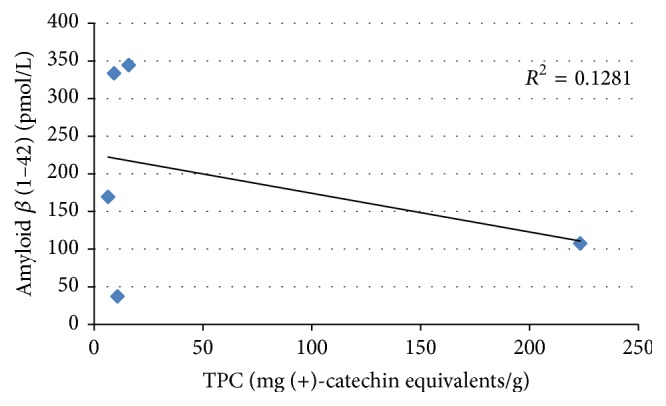
The correlation between levels of A*β* (1–42) and total phenolic contents of aqueous extracts from the sprouts of five plants.

**Figure 4:**
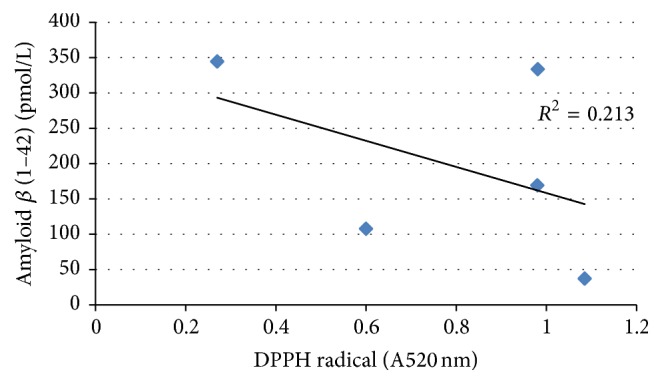
The correlation between levels of A*β* (1–42) and DPPH radical scavenging activities of aqueous extracts from the sprouts of five plants.

On the other hand, the correlations between A*β* (1–42) levels and TPC and DPPH radical scavenging activity were analyzed (Figures 3 and 4). As shown in Figure 3, the A*β* (1–42) levels were not observed to increase depending on TPC levels in PSAE. Specifically, no significant correlation (*r* = 0.358) was observed between A*β* (1–42) levels and TPC among our experimental samples. As shown in Figure 4, the DPPH radical scavenging activity of the 5 PSAE did not significantly correlate with A*β* (1–42) levels. The correlation coefficient for A*β* (1–42) levels was found to be smaller than 0.5 (*r* = 0.462) which proved that the DPPH radical scavenging activity could not be attributed to the A*β* (1–42) levels of the 5 PSAE. These results proved that the TPC and DPPH radical scavenging activity of these plant sprouts could not be clearly attributed to their A*β*-inhibition potential.

### 3.4. Effects of PSAE on D-Ribose Induced Glycation of BSA or LAB

**Figure 5:**
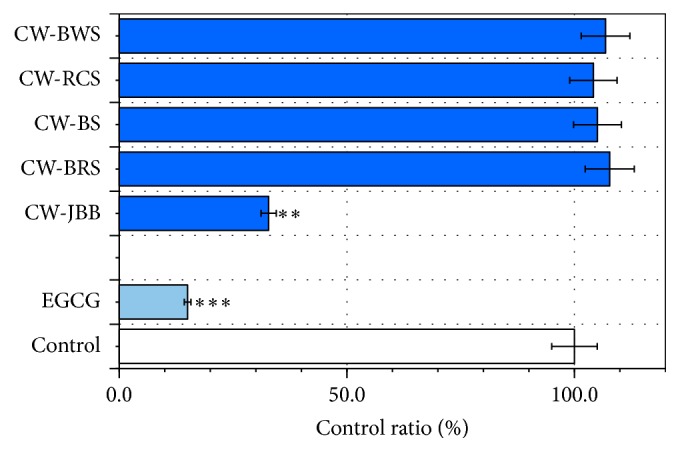
Changes in the fluorescence of BSA+D-ribose treated with plant sprouts' aqueous extracts (PSAE). BSA (final concentration 10 mg/mL) in the presence of D-ribose (final concentration 1 M) was kept at 37°C in Tris-HCl buffer (pH 7.4). PSAE was mixed with samples of BSA+D-ribose for up to 24 h. The fluorescence intensity of glycation was recorded (*λ*_ex_ 360 nm; *λ*_em_ 465 nm). BSA (or LAB) and D-ribose were used as a control. Aliquots were taken for measurements of fluorescence spectra (*λ*_ex_ = 360 nm). Values are mean ± SD of the three measurements. ^*∗∗∗*^ *P* < 0.001 and ^*∗∗*^ *P* < 0.01 compared with the controls.

**Figure 6:**
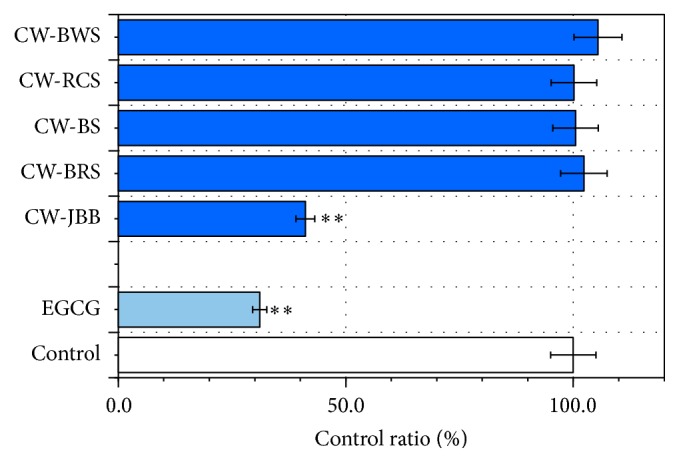
Changes in the fluorescence of LAB+D-ribose treated with plant sprouts' aqueous extracts (PSAE). LAB (final concentration 10 mg/mL) in the presence of D-ribose (final concentration 1 M) was kept at 37°C in Tris-HCl buffer (pH 7.4). PSAE was mixed with samples of LAB+D-ribose for up to 24 h. The fluorescence intensity of glycation was recorded (*λ*_ex_ 360 nm; *λ*_em_ 465 nm). LAB and D-ribose were used as a control. Aliquots were taken for measurements of fluorescence spectra (*λ*_ex_ = 360 nm). Values are mean ± SD of the three measurements. ^*∗∗*^ *P* < 0.01 compared with the controls.

In the present study, we investigated the effects of PSAE on glycation of BSA or LAB induced by D-ribose. The mixture samples were incubated at 37°C for up to 24 h (Figures 5 and 6). Inhibition of glycation was recognized in CW-JBB in BSA and LAB. Among the 5 PSAE, CW-JBB treatment demonstrated the strongest antiglycation potential. EGCG (100 *μ*M) treatment exhibited the greatest potential for antiglycation. In Figure 5, fluorescence assay results showed that BSA glycation levels did not decrease in the 24 h PSAE-loaded treatments relative to the control. However, BSA glycation by CW-JBB and EGCG inhibited 67.2 ± 3.58% and 85.0 ± 4.55%, respectively (*P* < 0.01). Among the 5 PSAE, CW-JBB caused a maximum decrease in BSA glycation (Figure 5).

The correlations between BSA glycation inhibition and A*β* (1–42) levels were analyzed. The A*β* (1–42) levels of the 5 PSAE did not significantly correlate with BSA glycation inhibition. The correlation coefficient for BSA glycation inhibition was found to be smaller than 0.5 (*r* = 0.409) which proved that the A*β* (1–42) levels could not be attributed to BSA glycation inhibition.

Fluorescence assay results (Figure 6) showed that LAB glycation level did not decrease in the 24 h PSAE-loaded treatment relative to the control. However, LAB glycation by CW-JBB inhibited 58.9 ± 2.85% (*P* < 0.01). EGCG (100 *μ*M) treatment exhibited the greatest potential for antiglycation (68.9 ± 3.59%). The correlations between LAB glycation inhibition and A*β* (1–42) levels were analyzed. The A*β* (1–42) levels of the 5 PSAE did not significantly correlate with LAB glycation inhibition. The correlation coefficient for LAB glycation inhibition was found to be smaller than 0.5 (*r* = 0.429) which proved that the A*β* (1–42) levels could not be attributed to the LAB glycation inhibition of the 5 PSAE. These results proved that the A*β* (1–42) levels of these plants could not be clearly attributed to their LAB glycation inhibition.

### 3.5. Effects of PSAE on Aggregates of D-Ribose-Glycated BSA or LAB

**Figure 7:**
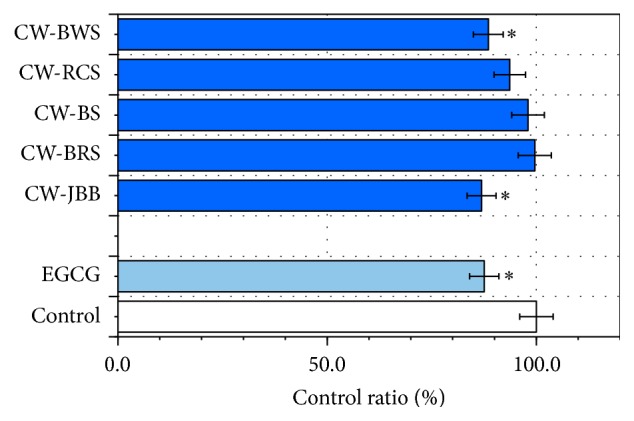
Changes in the Thioflavin T fluorescence of BSA+D-ribose treated with plant sprouts' aqueous extracts (PSAE). BSA (final concentration 10 mg/mL) in the presence of D-ribose (final concentration 1 M) was kept at 37°C in Tris-HCl buffer (pH 7.4). Thioflavin T (final concentration 30 *μ*M) was mixed with samples of BSA+D-ribose+PSAE, as described in Section 2.7. The fluorescence intensity of Thioflavin T was recorded (*λ*_ex_ 430 nm; *λ*_em_ 465 nm). BSA and D-ribose were used as a control. Aliquots were taken for measurements of fluorescence spectra (*λ*_ex_ = 430 nm). Values are mean ± SD of the three measurements. ^*∗*^ *P* < 0.05 compared with the controls.

We added ThT (a fluorescent reagent) to test whether PSAE is an inhibitor of amyloid-like aggregates (Figure 7). Fluorescence of ThT at 465 nm significantly increased in the presence of BSA incubated with D-ribose for 24 h. Fluorescence intensity showed about 36548 ± 1288 counts in BSA+D-ribose (Figure 7). Inhibition of aggregates was recognized in CW-BWS in BSA and CW-JBB in BSA and LAB. Among the 5 PSAE, CW-JBB treatment demonstrated the strongest antiaggregation potential. EGCG (100 *μ*M) treatment exhibited the second greatest potential for antiaggregation. In Figure 7, fluorescence assay results showed that BSA aggregation levels did not decrease in the 24 h PSAE-loaded treatments relative to the control. However, BSA aggregation by CW-JBB and CW-BWS inhibited 13.1 ± 1.15% and 11.5 ± 0.85%, respectively (*P* < 0.05). EGCG (100 *μ*M) treatment exhibited the second greatest potential for antiaggregation (12.5 ± 1.38%). BSA+D-ribose incubated with CW-JBB and CW-BWS showed decreases in ThT fluorescence under our experimental conditions.

The correlations between BSA aggregation inhibition and A*β* (1–42) levels were analyzed. The A*β* (1–42) levels of the 5 PSAE did not significantly correlate with BSA aggregation inhibition. The correlation coefficient for BSA aggregation inhibition was found to be smaller than 0.2 (*r* = 0.176) which proved that the A*β* (1–42) levels could not be attributed to BSA aggregation inhibition.

**Figure 8:**
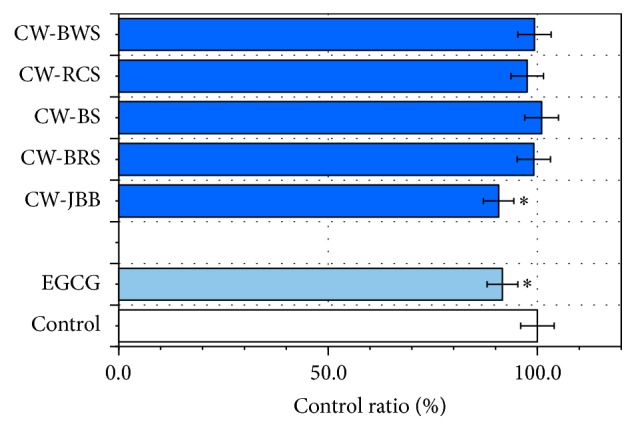
Changes in the Thioflavin T fluorescence of LAB+D-ribose treated with plant sprouts' aqueous extracts (PSAE). LAB (final concentration 10 mg/mL) in the presence of D-ribose (final concentration 1 M) was kept at 37°C in Tris-HCl buffer (pH 7.4). Thioflavin T (final concentration 30 *μ*M) was mixed with samples of LAB+D-ribose+PSAE, as described in Section 2.7. The fluorescence intensity of Thioflavin T was recorded (*λ*_ex_ 430 nm; *λ*_em_ 465 nm). LAB and D-ribose were used as a control. Aliquots were taken for measurements of fluorescence spectra (*λ*_ex_ = 430 nm). Values are mean ± SD of the three measurements. ^*∗*^ *P* < 0.05 compared with the controls.

Again, we added ThT to test whether PSAE is an inhibitor of amyloid-like aggregates (Figure 8). This time, however, fluorescence of ThT at 465 nm did not significantly decrease in the presence of LAB incubated with D-ribose for 24 h except for CW-JBB. Fluorescence intensity showed about 35751 ± 1276 counts in LAB+D-ribose. LAB+D-ribose incubated with PSAE except for CW-JBB showed no significant changes in ThT fluorescence under our experimental conditions. However, LAB+D-ribose incubated with CW-JBB showed significant decrease in ThT fluorescence (10.0 ± 0.43%). EGCG (100 *μ*M) treatment exhibited the second greatest potential for antiaggregation (9.6 ± 0.45%). The correlations between LAB aggregation inhibition and A*β* (1–42) levels were analyzed. The A*β* (1–42) levels of the 5 PSAE did not significantly correlate with LAB aggregation inhibition. The correlation coefficient for LAB aggregation inhibition was found to be smaller than 0.5 (*r* = 0.452) which proved that the A*β* (1–42) levels could not be attributed to LAB aggregation inhibition.

### 3.6. Congo Red (CR) Assay

**Figure 9:**
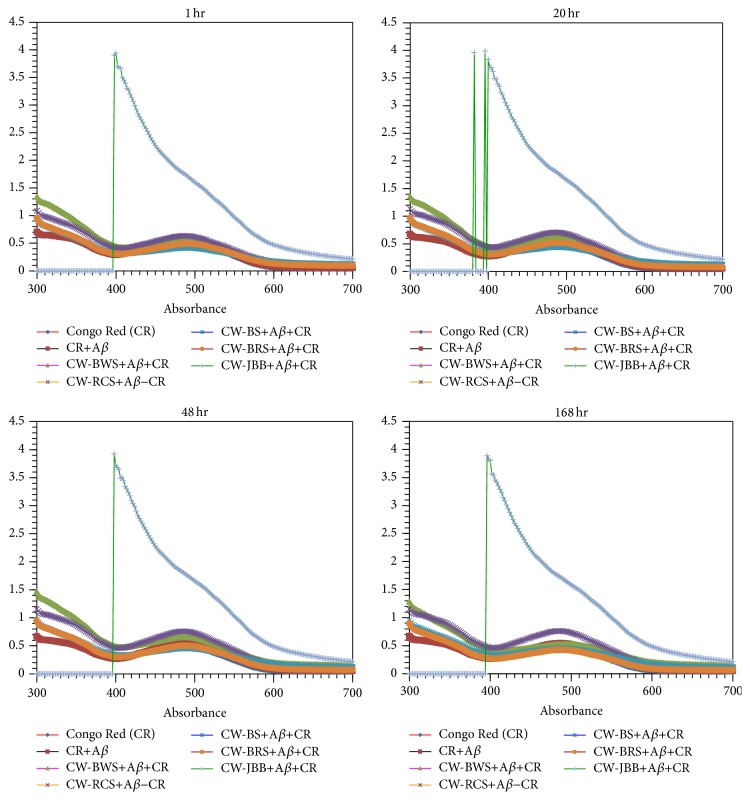
Absorption spectra of 5.0 × 10^−6^ M Congo red with and without plant sprouts' aqueous extracts (PSAE). The colour lines indicate the difference spectrum. The binding of CR was monitored using absorption spectroscopy for up to 168 h.

CR is a diazo dye that is widely used to identify amyloid deposits because of its ability to bind preferentially to the aggregated amyloid peptides [23]. We performed CR and A*β* binding experiments to determine whether PSAE are the modification of amyloid structures. CR and A*β* binding is detected as a red shift in the absorbance spectrum (shown by Table 3 and Figure 9). Figure 9 (1, 20, 48, and 168 hr) shows the absorption spectrum of CR and A*β* in the presence of PSAE. A significant red shift from 482 nm to 494 nm occurred upon binding of the dye to A*β* in 168 hr (Table 3 and Figure 9, 168 hr). Similarly, a significant red shift from 482 to 494 nm occurred upon binding of the dye to A*β* in the presence of CW-BWS. On the other hand, a red shift from 482 to 490 nm occurred upon binding of the dye to A*β* in the presence of CW-BS or CW-BRS. However, CW-RCS and A*β* mixture (484 nm) hardly influenced the absorption spectrum peak of CR. Namely, CR and A*β* binding in the presence of CW-RCS was not detected as a red shift in its absorbance spectrum. CW-JBB did not show a peak of the absorption spectrum.

The correlations between the absorption spectrum peaks of CR and A*β* (1–42) levels were analyzed. The A*β* (1–42) levels of the 4 (except for JBB) PSAE significantly correlate with the absorption spectrum peak of CR (Table 3). The correlation coefficient for the absorption spectrum peak of CR was found to be bigger than 0.8 (*r* = 0.882) which proved that the A*β* (1–42) levels could be attributed to the absorption spectrum peak of CR.

## 4. Discussion

In the present study, we investigated the effects of PSAE on A*β* (1–42) structure modification and demonstrated that treatment with PSAE effectively decreases A*β* (1–42) concentration (Figure 2).

Alzheimer's disease (AD) is receiving attention as “type 3 diabetes” and it is evident that this neurodegenerative disease has multiple shared pathologies with diabetes mellitus. Recently, we published two papers describing the utilization of plant extracts for improved treatment of diabetic complications [24, 25]. We investigated the impact of methanolic extracts from edible plants on the receptor for advanced glycation end products (RAGE) and endogenous secretory receptor for advanced glycation end products (esRAGE) production in human umbilical vein endothelial cells cultured in high glucose (4.5 g/L). The results showed that several extracts reduced RAGE production, and several extracts showed an increase in esRAGE. RAGE-mediated signaling pathway is related to A*β*-induced pathogenic responses [26]. Also, Yamagishi and Matsui indicated that V1- and C1-type domains of RAGE have a net positive charge that might act as an electrostatic trap for negatively charged macromolecules such as AGEs, high-mobility group protein box-1, S-100 calcium-binding protein, and amyloid *β* protein [27]. Li et al. described that the glycated amyloid *β* is more toxic [28]. Therefore, inhibition of glycation could be a molecular target for life-threatening disorders such as AD.

On the other hand, the protective effects of sprouts extract and its constituents against type 2 diabetes were reported. For instance, sunflower sprouts possess antiglycative and antioxidant activity [29], and broccoli sprouts powder could improve serum triglycerides and oxidized LDL/LDL-cholesterol ratio in type 2 diabetes [30]. Mung bean sprout extracts exerted an antidiabetic effect in diabetic KK-Ay mice [31], chickpea sprout was found to ameliorate some hyperglycemic symptoms of the diabetic rats, that is, reducing impairment of diabetic related spatial learning and memory [32], and Japanese radish sprout had a hypoglycemic activity in both normal and diabetic rats [33]. However, there is no article related to A*β* protein and glycation by plant sprouts. Therefore, we furthered this research by examining sprouts of plants, to determine whether or not they also exhibit the anti-A*β* activity and antiglycation.

A full understanding of the pathogenesis of AD has remained elusive, and an increasing amount of evidence is confirming that AD is a disease with numerous contributing factors, both genetic and environmental. It has been proposed that a chemical process known as glycation may contribute to both extensive protein cross-linking and oxidative stress in AD [34]. Glycation is the reaction of reducing sugars to proteins and lipids, resulting in protein modification. Glycation reactions are also elevated during metabolic dysfunction. Nonenzymatic protein glycation is an endogenous process in which reducing sugars react with amino groups in proteins through a series of Maillard reactions forming reversible Schiff-base and Amadori compounds. Wu et al. [35] show that the Schiff base is oxidized in the first stage of glycation and easily produces free radicals. These reactions increase the misfolding of proteins such as A*β* in AD. Thus, glycation is linked to metabolic dysfunction and may have a causal role in AD.

Taking the statement above into account, we examined the glycation inhibition ability of PSAE. In this study, we used the modification model of BSA and D-ribose by Wei et al. [10]. We considered whether PSAE might be inhibiting BSA or LAB glycation. Based on its fluorescence properties, we studied the influence of PSAE on the glycation of D-ribose with BSA or LAB. Our results demonstrated that CW-JBB in BSA and LAB inhibited glycation of BSA or LAB.

As a result, we found that PSAE except in CW-JBB did not inhibit glycation of BSA (Figure 5). A similar result was provided for the different protein, LAB (Figure 6). Significant results were evident in BSA or LAB aggregation except in CW-BWS in BSA and CW-JBB in BSA and LAB (Figures 7 and 8). CW-JBB had the ability to inhibit glycation and also the ability to inhibit aggregation of the protein. In other words, reactions to protein and sugar were inhibited by CW-JBB, and CW-JBB inhibited aggregation of the cross-linked, structure producing protein. Our results agreed with our prediction. It is thought likely that, in the stage before protein is modified by sugar, CW-JBB binds to the protein and disturbs protein modification by sugar. Namely, we propose that the sugar-protein binding site may also be the binding site for the CW-JBB protein and that CW-JBB may compete with D-ribose.

The process of BSA glycation is triggered by the production of an irreversible heterologous byproduct. Bhattacherjee and Chakraborti [36] reported that the extract of* Piper betle* Linn. leaf may have beneficial effect in preventing protein glycation, and considering its relative amounts present in the extract, rutin appeared to be the most active antiglycating agent. On the other hand, rutin was isolated from flower buds of Japanese butterbur (*Petasites japonicus* subsp.* gigantea* Kitam.) [16]. Therefore, the likelihood that rutin occurs in CW-JBB is quite high. As for the result of 24 h incubation under the coexistence of BSA and D-ribose (Figure 5), CW-JBB is thought to have produced the result that contributed to the carbonyl formation of protein, as the extract of* Piper betle* Linn. leaf controlled the reaction. Therefore, because we have shown it is possible that CW-JBB competes with sugar in protein modification, CW-JBB may contribute to a meaningful delay in the pathological progress of diseases such as AD. To examine the effects of PSAE on the A*β* (1–42) levels, PSAE+A*β* samples were analyzed by A*β* ELISA. We demonstrated that treatment with PSAE effectively decreases A*β* levels (Figure 2). The above data (Figures 2, 5, and 6) show that CW-JBB induced the inhibition of both A*β* (1–42) levels and protein glycation. Notably, CW-JBB may reduce A*β* (1–42) levels by affecting protein conformation. Whereas Fu et al. [37] reported that curcumin and resveratrol bind to the N-terminus of A*β*42 monomers and cap the height of the oligomers, Feng et al. also described that resveratrol may directly bind to A*β*42, interfere in A*β*42 aggregation, and change the A*β*42 oligomer conformation [38]. However, in this case, resveratrol could not prevent A*β*42 oligomer formation.

Our data indicated that the A*β* (1–42) levels of the 4 (except for JBB) PSAE significantly correlate with the absorption spectrum peak of CR. We showed here that the correlation coefficient for the absorption spectrum peak of CR was found to be bigger than 0.8 (*r* = 0.882) which proved that the A*β* (1–42) levels could be attributed to the absorption spectrum peak of CR. And, no band of A*β* monomer (4.5 kDa) was recognized in CW-JBB. Similarly, the A*β* monomer (4.5 kDa) band became light in CW-RCS, CW-BS, CW-BRS, and CW-BWS. Thus, the mechanism that decreased a level of amyloid *β* may be modification by PSAE. It might act in similar ways to curcumin, resveratrol, and* Ginkgo biloba* extract.

On the other hand, Yao et al. showed that the* Ginkgo biloba* extract EGb 761 rescues PC12 neuronal cells from A*β*-induced cell death by inhibiting the formation of A*β*-derived diffusible neurotoxic ligands [39]. Addition of the extract of* Ginkgo biloba* leaves, EGb 761, in combination with the A*β* protein prevented, in a dose-dependent manner, A*β*-induced free radical production and cell death. These results indicate that the terpenoid and flavonoid constituents of EGb 761 are responsible for rescuing the neuronal cells from A*β*-induced cell death, their mechanism of action being distinct from their antioxidant properties. Because pre- and posttreatment with EGb 761 did not protect the cells from A*β*-induced neurotoxicity, they examined whether EGb 761 interacts directly with A*β*.* In vitro* reconstitution studies demonstrated that EGb 761 inhibits the formation of A*β*-derived diffusible neurotoxic soluble ligands, suggested to be involved in the pathogenesis of AD. We also indicated that the DPPH radical scavenging activity of the 5 PSAE did not significantly correlate with A*β* (1–42) levels. Based upon the foregoing, the PSAE mechanism may be the same as that of EGb 761. PSAE may interact with A*β*. In addition, there were reports [12, 13, 16] revealing the presence of terpenoids or flavonoids in water soluble extract of sprout (Table 2). For example, it is known that buckwheat sprouts contain several flavonoids, including orientin, isoorientin, vitexin, isovitexin, rutin, and quercetrin [12]. Judging from the mechanism of* Ginkgo biloba* extract, it is likely that PSAE also contains a terpenoid and a flavonoid and might act in similar ways to* Ginkgo biloba* extract.

This study found that treatment with PSAE effectively decreases A*β* concentration, showing promise in the development of a novel and functional food for suppressing AD.

## 5. Conclusion

The neuroprotective effects of PSAE were evaluated by measuring levels of A*β* in mixtures (A*β*  and PSAE) with A*β* ELISA assay. The results demonstrate that the treatment with PSAE decreased A*β* levels. The highest value was observed with CW-RCS and A*β* mixture. Furthermore, we performed CR and A*β* binding experiments to determine whether PSAE are the modification of amyloid structures. CW-RCS and A*β* mixture did not affect the CR spectrum. Namely, CR binding in CW-RCS and A*β* mixture was not detected as a red shift in its absorbance spectrum. And, the A*β* (1–42) levels of the 4 (except for JBB) PSAE significantly correlate with the absorption spectrum peak of CR. The mechanism that decreased a level of A*β* may be modification by PSAE. It might act in similar ways to curcumin, resveratrol, and* Ginkgo biloba* extract.

## Figures and Tables

**Table 1:** Extract concentrations from plant sprouts. We selected five plant sprouts (buckwheat sprout (BWS), red cabbage sprout (RCS), broccoli sprout (BS), brussels sprout (BRS), and Japanese butterbur sprout flower buds (JBB)). Plant sprouts (100 g) were minced into 5 mm fragments and extracts were obtained with cold water (CW, 20°C), hot water (HW, 100°C, 10 min), and methanol (MH) overnight at room temperature. Extract concentrations were expressed as gram of dry weight/mL of extract volume. The concentration of plant sprouts' extracts (PSE) was calculated as catechin equivalents per gram of plant sprouts. ^*∗*^Means ± standard deviation (SD) of three independent experiments.

Extract	Initial dry weight (g)^**∗**^	Extract volume (mL)^*∗*^	Extract concentration (g of dry weight/mL of extract volume)^**∗**^	Total phenolic content (mg of catechin equiv/g)^**∗**^
CW-BWS	8.40 ± 0.53	2.90 ± 0.45	2.90 ± 0.53	15.9 ± 0.37
CW-RCS	4.87 ± 0.45	2.50 ± 0.38	1.95 ± 0.34	10.8 ± 0.41
CW-BS	5.43 ± 0.52	2.50 ± 0.31	2.17 ± 0.32	6.5 ± 0.21
CW-BRS	10.4 ± 0.65	9.20 ± 0.63	1.13 ± 0.17	9.2 ± 0.71
CW-JBB	10.0 ± 0.65	11.5 ± 0.55	0.87 ± 0.08	223.3 ± 7.37
HW-BWS	8.40 ± 0.45	4.30 ± 0.35	1.95 ± 0.13	339.1 ± 8.37
HW-RCS	4.87 ± 0.25	3.20 ± 0.28	1.52 ± 0.22	36.1 ± 1.41
HW-BS	5.43 ± 0.35	5.50 ± 0.31	0.99 ± 0.22	242.1 ± 6.21
HW-BRS	10.0 ± 0.55	7.10 ± 0.46	1.41 ± 0.27	76.5 ± 3.71
HW-JBB	8.48 ± 0.45	7.50 ± 0.45	1.13 ± 0.36	397.8 ± 9.37
MH-BWS	10.0 ± 0.55	3.95 ± 0.25	2.53 ± 0.39	433.1 ± 9.37
MH-RCS	10.0 ± 0.47	3.30 ± 0.28	3.03 ± 0.34	235.2 ± 5.41
MH-BS	10.0 ± 0.45	3.30 ± 0.11	3.03 ± 0.22	241.0 ± 5.21
MH-BRS	10.0 ± 0.48	3.20 ± 0.16	3.13 ± 0.27	317.3 ± 7.71
MH-JBB	37.2 ± 1.05	12.2 ± 0.56	3.05 ± 0.38	371.0 ± 8.37

**Table 2:** Bioactive compounds of sprouts or matured plants. We selected five plant sprouts (buckwheat (BW) sprout (BWS), red cabbage (RC) sprout (RCS), broccoli (B) sprout (BS), brussels (BR) sprout (BRS), and Japanese butterbur sprout flower buds (JBB)). This table shows the reported bioactive compounds of sprouts or matured plants.

Samples name	Compounds	References
BWS or BW	Quercetin glucoside	[11]

RCS or RC	Orientin, isoorientin, vitexin, isovitexin, rutin, and quercetin	[12]
	Kaempferol, quercetin derivatives, hydroxybenzoic acid, and cyanidin	[13]

BS or B	Quercetin and sulforaphane	[14]
	Sinigrin	[15]

BRS or BR	Kaempferol and quercetin derivatives	[13]
	Hydroxybenzoic acid and sinigrin	[15]

JBB (flower buds) or JBB	Quercetin glucoside	[16]
	Petasiphenol	[17]
	Fukinolic acid	[18]
	Triterpene glycosides	[19]

**Table 3:** Absorption spectrum peak determined using Congo Red (CR) assay by spectrophotometry in plant sprouts' aqueous extracts (PSAE). We selected five plant sprouts (buckwheat sprout (BWS), red cabbage sprout (RCS), broccoli sprout (BS), brussels sprout (BRS), and Japanese butterbur sprout flower buds (JBB)). This table shows the absorption spectrum peak of CR and A*β* (1–42). The binding of CR and A*β* was monitored using absorption spectroscopy. Values are the means, *n* = 3.

Samples	Absorbance peak
CW-BWS+A*β*+CR	494
CW-RCS+A*β*-CR	484
CW-BS+A*β*+CR	490
CW-BRS+A*β*+CR	490
CW-JBB+A*β*+CR	None

CR+A*β*	494
Congo Red (CR)	482

## References

[1] Angeloni C., Zambonin L., Hrelia S. (2014). Role of methylglyoxal in alzheimer's disease. *BioMed Research International*.

[2] Guo L., Yang R., Wang Z., Guo Q., Gu Z. (2014). Effect of NaCl stress on health-promoting compounds and antioxidant activity in the sprouts of three broccoli cultivars. *International Journal of Food Sciences and Nutrition*.

[3] Chiavaro E., Mazzeo T., Visconti A., Manzi C., Fogliano V., Pellegrini N. (2012). Nutritional quality of sous vide cooked carrots and brussels sprouts. *Journal of Agricultural and Food Chemistry*.

[4] Jaiswal A. K., Rajauria G., Abu-Ghannam N., Gupta S. (2011). Phenolic composition, antioxidant capacity and antibacterial activity of selected Irish Brassica vegetables. *Natural Product Communications*.

[5] Shetty K., Wahlqvist M. L. (2004). A model for the role of the proline-linked pentose-phosphate pathway in phenolic phytochemical bio-synthesis and mechanism of action for human health and environmental applications. *Asia Pacific Journal of Clinical Nutrition*.

[6] Hoelzl C., Bichler J., Ferk F. (2005). Methods for the detection of antioxidants which prevent age related diseases: a critical review with particular emphasis on human intervention studies. *Journal of Physiology and Pharmacology*.

[7] Złotek U., Świeca M., Jakubczyk A. (2014). Effect of abiotic elicitation on main health-promoting compounds, antioxidant activity and commercial quality of butter lettuce (Lactuca sativa L.). *Food Chemistry*.

[8] Schalkwijk C. G., Stehouwer C. D. A., van Hinsbergh V. W. M. (2004). Fructose-mediated non-enzymatic glycation: sweet coupling or bad modification. *Diabetes/Metabolism Research and Reviews*.

[9] Yamagishi S.-I. (2012). Potential clinical utility of advanced glycation end product cross-link breakers in age- and diabetes-associated disorders. *Rejuvenation Research*.

[10] Wei Y., Chen L., Chen J., Ge L., He R. Q. (2009). Rapid glycation with D-ribose induces globular amyloid-like aggregations of BSA with high cytotoxicity to SH-SY5Y cells. *BMC Cell Biology*.

[11] Muhammad S., Fatima N. (2015). In silico analysis and molecular docking studies of potential angiotensin-converting enzyme inhibitor using quercetin glycosides. *Pharmacognosy Magazine*.

[12] Nam T. G., Lee S. M., Park J. H., Kim D. O., Baek N. I., Eom S. H. (2015). Flavonoid analysis of buckwheat sprouts. *Food Chemistry*.

[13] Duchnowicz P., Bors M., Podsedek A., Koter-Michalak M., Broncel M. (2012). Effect of polyphenols extracts from *Brassica* vegetables on erythrocyte membranes (*in vitro* study). *Environmental Toxicology and Pharmacology*.

[14] Lozanovski V. J., Houben P., Hinz U., Hackert T., Herr I., Schemmer P. (2014). Pilot study evaluating broccoli sprouts in advanced pancreatic cancer (POUDER trial)—study protocol for a randomized controlled trial. *Trials*.

[15] Awasthi S., Saraswathi N. (2016). Sinigrin, a major glucosinolate from cruciferous vegetables restrains non-enzymatic glycation of albumin. *International Journal of Biological Macromolecules*.

[16] Matsuura H., Amano M., Kawabata J., Mizutani J. (2002). Isolation and measurement of quercetin glucosides in flower buds of Japanese butterbur (*Petasites japonicus* subsp. *gigantea* Kitam.). *Bioscience, Biotechnology and Biochemistry*.

[17] Mizushina Y., Kamisuki S., Kasai N. (2002). Petasiphenol: a DNA polymerase *λ* inhibitor. *Biochemistry*.

[18] Hasa Y., Tazaki H. (2004). Biosynthesis of fukinolic acid isolated from *Petasites japonicus*. *Bioscience, Biotechnology and Biochemistry*.

[19] Shimoda H., Tanaka J., Yamada E., Morikawa T., Kasajima N., Yoshikawa M. (2006). Anti type I allergic property of Japanese butterbur extract and its mast cell degranulation inhibitory ingredients. *Journal of Agricultural and Food Chemistry*.

[20] Okada Y., Okada M. (2014). Komatsuna seed extracts protection against amyloid *β*(1-42)-induced neuronal cell death. *Journal of Diabetes & Metabolism*.

[21] Gao X., Ohlander M., Jeppsson N., Björk L., Trajkovski V. (2000). Changes in antioxidant effects and their relationship to phytonutrients in fruits of sea buckthorn (*Hippophae rhamnoides* L.) during maturation. *Journal of Agricultural and Food Chemistry*.

[22] Negro C., Tommasi L., Miceli A. (2003). Phenolic compounds and antioxidant activity from red grape marc extracts. *Bioresource Technology*.

[23] Chen J., Armstrong A. H., Koehler A. N., Hecht M. H. (2010). Small molecule microarrays enable the discovery of compounds that bind the Alzheimer's A*β* peptide and reduce its cytotoxicity. *Journal of the American Chemical Society*.

[24] Okada M., Okada Y. (2015). Effects of methanolic extracts of edible plants on RAGE in high-glucose-induced human endothelial cells. *Bio-Medical Materials and Engineering*.

[25] Okada Y., Okada M. (2015). Effects of methanolic extracts from edible plants on endogenous secretory receptor for advanced glycation end products induced by the high glucose incubation in human endothelial cells. *Journal of Pharmacy and Bioallied Sciences*.

[26] Lv C., Wang L., Liu X. (2015). Multi-faced neuroprotective effects of geniposide depending on the RAGE-mediated signaling in an Alzheimer mouse model. *Neuropharmacology*.

[27] Yamagishi S.-I., Matsui T. (2015). Role of receptor for advanced glycation end products (RAGE) in liver disease. *European Journal of Medical Research*.

[28] Li X.-H., Du L.-L., Cheng X.-S. (2013). Glycation exacerbates the neuronal toxicity of *β*-amyloid. *Cell Death and Disease*.

[29] Sun Z., Chen J., Ma J. (2012). Cynarin-rich sunflower (*Helianthus annuus*) sprouts possess both antiglycative and antioxidant activities. *Journal of Agricultural and Food Chemistry*.

[30] Bahadoran Z., Mirmiran P., Hosseinpanah F., Rajab A., Asghari G., Azizi F. (2012). Broccoli sprouts powder could improve serum triglyceride and oxidized LDL/LDL-cholesterol ratio in type 2 diabetic patients: a randomized double-blind placebo-controlled clinical trial. *Diabetes Research and Clinical Practice*.

[31] Yao Y., Chen F., Wang M., Wang J., Ren G. (2008). Antidiabetic activity of Mung bean extracts in diabetic KK-Ay mice. *Journal of Agricultural and Food Chemistry*.

[32] Mao X., Zhang L., Xia Q. (2008). Vanadium-enriched chickpea sprout ameliorated hyperglycemia and impaired memory in streptozotocin-induced diabetes rats. *BioMetals*.

[33] Taniguchi H., Kobayashi-Hattori K., Tenmyo C. (2006). Effect of Japanese radish (*Raphanus sativus*) sprout (Kaiware-daikon) on carbohydrate and lipid metabolisms in normal and streptozotocin-induced diabetic rats. *Phytotherapy Research*.

[34] Fawver J. N., Schall H. E., Petrofes Chapa R. D., Zhu X., Murray I. V. J. (2012). Amyloid-*β* Metabolite sensing: biochemical linking of glycation modification and misfolding. *Journal of Alzheimer's Disease*.

[35] Wu C.-H., Huang S.-M., Lin J.-A., Yen G.-C. (2011). Inhibition of advanced glycation endproduct formation by foodstuffs. *Food and Function*.

[36] Bhattacherjee A., Chakraborti A. S. (2013). Inhibitory effect of Piper betle Linn. leaf extract on protein glycation—quantification and characterization of the antiglycation components. *Indian Journal of Biochemistry and Biophysics*.

[37] Fu Z., Aucoin D., Ahmed M., Ziliox M., Van Nostrand W. E., Smith S. O. (2014). Capping of A*β*42 oligomers by small molecule inhibitors. *Biochemistry*.

[38] Feng Y., Wang X.-P., Yang S.-G. (2009). Resveratrol inhibits beta-amyloid oligomeric cytotoxicity but does not prevent oligomer formation. *NeuroToxicology*.

[39] Yao Z.-X., Drieu K., Papadopoulos V. (2001). The *Ginkgo biloba* extract EGb 761 rescues the PC12 neuronal cells from *β*-amyloid-induced cell death by inhibiting the formation of *β*-amyloid-derived diffusible neurotoxic ligands. *Brain Research*.

